# Learning by teaching basic life support: a non-randomized controlled trial with medical students

**DOI:** 10.1186/s12909-019-1500-7

**Published:** 2019-03-01

**Authors:** Sérgio Geraldo Veloso, Gabriel Santos Pereira, Nathália Nascimento Vasconcelos, Maria Helena Senger, Rosa Malena Delbone de Faria

**Affiliations:** 1 Departamento de Medicina, Curso de Medicina, Universidade Federal de São João del Rei, Praça Dom Helvécio, 74 - Dom Bosco, São João del Rei, MG 36301-160 Brazil; 2 Faculdade de Ciências Médicas e da Saúde, Pontifícia Universidade Católica de São Paulo, Sorocaba, SP Brazil; 3 Departamento de Propedêutica Complementar, Faculdade de Medicina – Universidade Federal de Minas Gerais, Belo Horizonte, MG Brazil

**Keywords:** Cardiopulmonary resuscitation, Basic life support, Active teaching methodologies, Meaningful learning, Medical education, Simulation, Social accountability

## Abstract

**Background:**

Cardiopulmonary resuscitation is usually taught in universities through theoretical lectures and simulations on mannequins with low retention of knowledge and skills. New teaching methodologies have been used to improve the learning, placing the student at the center of the process. Likewise, the outside community knows next to nothing about cardiopulmonary resuscitation. Patients who have an out-of-hospital cardiac arrest will die if the effective maneuvers are not promptly done. Learning by teaching could be a way to answer both requirements. It was therefore decided to evaluate whether the medical students’ cardiopulmonary resuscitation performance would improve when they teach other people, and if those people could learn with them effectively.

**Methods:**

A non-randomized controlled trial was designed to assess whether teaching Basic Life Support would increase students’ learning. Socially engaged, seeking to disseminate knowledge, 92 medical students were trained in Basic Life Support and who subsequently trained 240 community health professionals. The students performed theoretical and practical pre- and post-tests whereas the health professionals performed theoretical pre- and post-tests and one practical test. In order to assess the impact of teaching on students’ learning, they were divided into two groups: a case group, with 53 students, reassessed after teaching health professionals, and a control group, with 39 students, reassessed before teaching.

**Results:**

The practical students’ performance of the case group went from 13.3 ± 2.1 to 15.3 ± 1.2 (maximum = 17, *p* < 0.001) and theoretical from 10.1 ± 3.0 to 16.4 ± 1.7 (maximum = 20, p < 0.001) while the performance of the control group went from 14.4 ± 1.6 to 14.4 ± 1.4 (*p* = 0.877) and from 11.2 ± 2.6 to 15.0 ± 2.3 (p < 0.001), respectively. The theoretical performance of the health professionals changed from 7.9 ± 3.6 to 13.3 ± 3.2 (p < 0.001) and the practical performance was 11.7 ± 3.2.

**Conclusions:**

The students who passed through the teaching activity had a theoretical and practical performance superior to that of the control group. The community was able to learn from the students. The study demonstrated that the didactic activity can be an effective methodology of learning, besides allowing the dissemination of knowledge. The University, going beyond its academic boundaries, performs its social responsibility.

## Background

In Brazil, in recent years, a new governmental educational policy, called “More Doctors Program”, has sought to increase the number of doctors and their better distribution throughout the country, through rural medical schools [1, 2]. One of the stages of the process was the expansion of the number of places and the creation of new medical schools, moving away from the traditional Flexnerian teaching model [3, 4], through a great reform emphasizing active methodologies of teaching and learning [5], focusing on the students and at the service of the communities [6, 7]. In this scenario, in the implementation of one of these new medical schools, theoretical references were looked for that could support methodologies capable of increasing students’ acquisition, retention and transfer of knowledge, linked to the fulfillment of social demands [8]. Based on andragogy [9] the teaching-learning methodology [10–12] was chosen as a way to increase students’ knowledge within higher levels of the Miller pyramid [13], as well as to meet the needs of the local community.

One of the demands of the basic health unit professionals was an urgency and emergency training course. Due to the great practical repercussion and the availability of equipment (mannequins) at the university, cardiopulmonary resuscitation emerged as a course option [14–16]. In Brazil, the teaching of cardiopulmonary resuscitation techniques, Basic Life Support (BLS), has not traditionally been carried out during high school, only being taught at universities or vocational courses [17]. In the medical curriculum adopted for this new school, the BLS teaching takes place in the second term of the course, with the use of simulation, and the students will have another contact with it in the modules aimed at emergencies in the 8th and 11th terms, in practical scenarios. Some students, after the second period, begin to develop extension activities related to medical urgency and BLS.

Cardiopulmonary resuscitation is capable of enhancing people’s survival in dramatic cases, whose outcome would inevitably be death [18]. In cardiac arrest, the elapsed time is inversely proportional to the chance of survival [19]. On the other hand, cardiopulmonary resuscitation, if performed adequately [14, 19], has the potential to prolong the patient’s life, while waiting for adequate help to arrive,. There is evidence that community-based training in BLS, even in countries with less economic resources, is an effective intervention to improve public health, especially in ensuring the “chain-of-survival” [15].

Universalizing the cardiopulmonary resuscitation skills has great social relevance [20–22]. Medical students have already proved to be a group with great multiplier potential in disseminating BLS techniques to various social groups, such as secondary students, health professionals or the community in general [21, 23, 24]. The university, in the exercise of its social responsibility, must seek alternatives for continuing education, maximizing the performance of the students and the community [25, 26].

This study used the methodology of learning by teaching in cardiopulmonary resuscitation and assessed whether the students’ performance (knowledge and skills) would increase after going through the activity of teaching others, and at the same time, those skills could be disseminated within the community.

## Methods

Second-term medical students at a Brazilian university were trained in cardiopulmonary resuscitation techniques following BLS precepts. After that, they empowered community members by giving them a theoretical and simulated BLS course [21, 27, 28]. An initial pilot study demonstrated a very positive result [29].

**Fig. 1:**
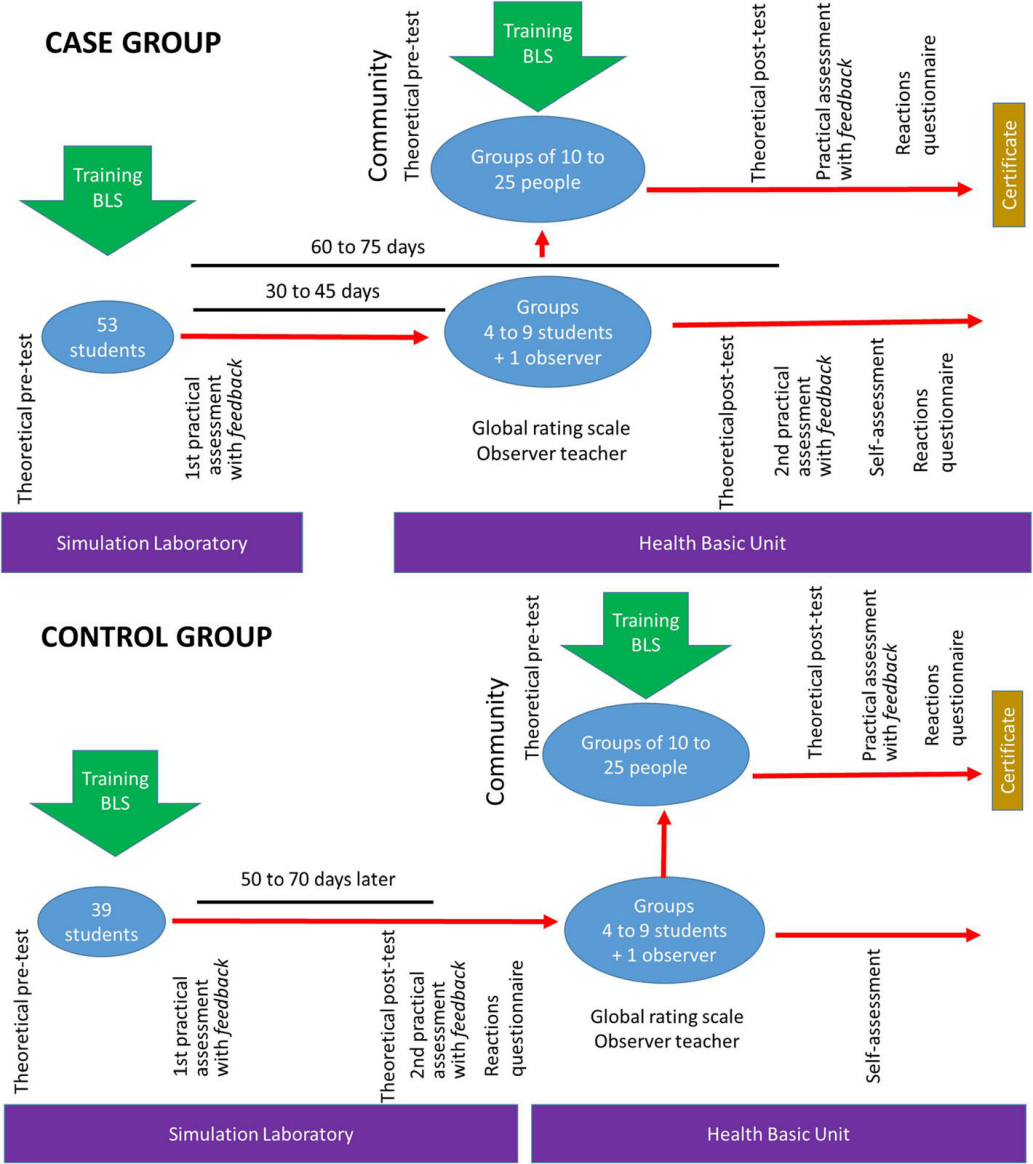
Illustrations of the different stages and assessments and their order applied to students (case and control groups) and community

A non-randomized controlled trial with case and control groups was designed, assessing students’ performance through theoretical (cognitive) and practical (skills) tests, pre- and post- type, in which the intervention was the act of teaching other people. In turn, the people who learned were also assessed (Fig. 1).

The inclusion criterion for the students was to be in the second term of the medical course. For the members of the community it was to work in the places where the students usually practice and also to volunteer to participate. Exclusion criteria were: less than 18 years of age, physical weakness (permanent or temporary) that compromised the performance of resuscitation maneuvers and illiteracy.

### Students

Initially, the students (from the case and control groups) performed a theoretical pre-test of 20 multiple choice items with five alternatives each, on BLS. This test was developed by a university professor with experience in Medical Emergency, certified by Advanced Cardiovascular Life Support (ACLS) as a provider, and then reviewed by another professor with similar experience and applied to volunteer students who did not take part in the project. Subsequently, the same test was applied to a group of students that constituted the pilot design of this study. All case and control students learned BLS and first aid according to the American Heart Association [14], by a teacher approved in the ACLS provider course. Each student attended 26 h of theoretical classes and simulations and took four hours of assessments. In the classroom, they clarified doubts and had access to the simulation laboratory. In the end, they performed the first practical assessment in which they were required to attend, as a single rescuer, a victim (a realistic simulation mannequin of medium fidelity) in cardiopulmonary arrest, with an automatic external defibrillator (AED) training and a ventilation bag-mask. The station had an average duration of seven minutes. The assessor, another teacher also approved in the ACLS, did not participate in the students’ training classes. The performance in the practical assessment was measured by a checklist consisting of 17 items [30] followed by feedback [31].

For the next stage, the students were divided into two groups: the case group (53 students from 3 classes) and the control group (39 students from 2 classes). The whole project took two and a half years and the five classes were divided, not randomly, into case-control-case-control-case, respectively. As the students’ activities were part of the curriculum, it was not possible to randomise the students into case and control in the same class because it could generate different conditions among learners in the summative assessment of the course.

### Case group

After 30 to 45 days of the first practical assessment, and with no other contact with BLS since then, the case group, subdivided into groups of four to nine students [32] taught BLS to groups of 10 to 25 people in the community in two-shift courses of four hours each, with two low-fidelity mannequins, one AED and two bag-mask devices. The students had autonomy in the preparation of the courses. They also prepared posters and booklets containing information on BLS and distributed them to the participants. An observational teacher followed and assessed the student activity (global rating scale) of 9 items, with answers on a Likert scale: from 0 to 10, assessing clarity and objectivity, self-confidence in the presentation, practical skills, ethics, care with course materials, teaching competence, interpersonal skills in relation to their peers, the community members and the basic health unit professionals [33].

The case group students, 15 to 30 days after the end of the course given (60 to 75 days after the first practical assessment), carried out the theoretical test of 20 items, the same as the pre-test, but with the items arranged in a different order, and the second practical assessment, the same as the first with checklist [30, 31]. Finally, they performed a self-assessment [34] about their performance in class and their performance in the teaching activity, with a total of 14 items being assessed on a Likert scale from 1 to 5.

The self-assessment and the global rating scale were each weighted out of 10 in order to make both the calculations and comparisons easier. Students and observer teachers were unaware of the case or control status of the classes.

### Control group

The control group received the same BLS teaching as the case group and were submitted to the theoretical pre-test and the first practical assessment [30, 31]. Then, they also prepared posters and booklets explaining BLS [10]. However, after 50 to 70 days of the first practical assessment, and without any other contact with BLS since the last assessment, a new theoretical assessment (theoretical post-test) and the second practical assessment were carried out. Both assessments were the same as the first, as in the case group. One week after this assessment, they taught BLS to the members of the community (crossover), being assessed by the observer teacher (global rating scale) [33]. After doing this, they performed the self-assessment [34], as in the case group.

All activities of the students were curricular educational activities being carried out within the school hours. All students performed satisfactorily within the educational module (> 60%). A single assessor followed all the practical assessments.

### Community (health professionals and laymen)

The community members consisted of health professionals (nurses, nursing technicians, community health agents, dentists, and ambulance drivers) together with laymen nominated by the local public health authority who were interested in attending the BLS courses provided by the students. These people were given the necessary guidance about the project before signing the free and informed consent form. The courses took place in two shifts of four hours, in two subsequent weeks, with a third shift for theoretical and practical assessment, totaling 10 h [27, 28]. These activities occurred, preferably, in the health units, during working hours as a training activity. They performed the theoretical pre-test of 20 items (the same of the students’). Then the courses started and on the third day, without the students’ presence, they carried out the assessments: theoretical post-test (the same as the pre-test, with the questions in a different order) and the only practical assessment (similar to the students’, with checklist) followed by feedback [30, 31]. The graduates of the course received a certificate from the university. The theoretical post-test and the practical assessment occurred between 10 and 20 days after the pre-test.

All participants were made aware of the research objectives and their stages, signing the consent form with full agreement. The assessments were individual, in reserved places, and the results were only known to the assessor and the individual being assessed, thereby ensuring confidentiality.

Data were analyzed using IBM SPSS Statistic version 20. All variables were tested for normality using the Shapiro-Wilk test, which is considered a robust test to determine whether the data are parametric or not. Parametric independent variables were compared by the Student’s T test (comparing the means of the independent groups) and the independent nonparametric variables by the Mann-Whitney test (comparing the medians of the independent groups). In the parametric paired variables, the paired Student’s T test was used (comparing the means of the dependent groups) and for the nonparametric related variables, the Wilcoxon signed-rank test was used (comparing the medians of the dependent groups). Statistically significant difference was considered when *p* < 0.05.

## Results

Ninety-two students from the second term of the medical course (mean age ± standard deviation: 20.4 ± 2.1 years, 34% men and 66% women) from five classes participated in at least one of the research stages. Three classes (53 students) constituted the case group (20.6 ± 2.4 years, 40% men and 60% women) and two classes (39 students) the control group (20.1 ± 1.7, 26% men and 74% women). Community members (*n* = 240; 40.9 ± 10.3 years, 29% men and 71% women) participated in at least one of the stages, of whom 65 were community health agents (27%), 60 nursing technicians (25%), 43 drivers (18%), 24 higher level professionals (nurses, dentists, social workers, psychologists and educators (10%) and 48 other middle and fundamental level professionals (20%).

The number of individuals varied in the different stages and assessments performed which occurred on different days. Despite that, 34 case group students, 35 control group students and 155 community members participated in all the stages and assessments.

The students’ performance in the case group was compared to that of the control group in the different assessments performed (Table 1).

**Table 1:** Comparison between students’ performance (case vs. control)

Assessment (maximum)	Group	n	x ± s	*p* value
Theoretical pre-test (20 marks)	Case	36	10,1 ± 2,9	0,137^a^
	Control	37	11,1 ± 2,6	
Theoretical post-test (20 marks)	Case	*34*	*16,4 ± 1,7*	*0,005* ^*a*^
	Control	*37*	*15,0 ± 2,3*	
1st practical assessment (17 marks)	Case	53	13,4 ± 2,1^c^	0,050^b^
	Control	38	14,2 ± 1,6^c^	
2nd practical assessment (17 marks)	Case	*50*	*15,3 ± 1,2* ^*c*^	*0,001* ^*b*^
	Control	36	*14,4 ± 1,4* ^*c*^	
Self-assessment (10 marks)	Case	50	8,6 ± 0,8^c^	0,671^b^
	Control	37	8,6 ± 0,6	
Global rating scale (10 marks)	Case	53	9,4 ± 0,6^c^	0,086^b^
	Control	36	9,1 ± 0,6	

x ± s – mean ± standard deviation

^a^Student’s T test

^b^Mann Whitney test

^c^Nonparametric

**Fig. 2:**
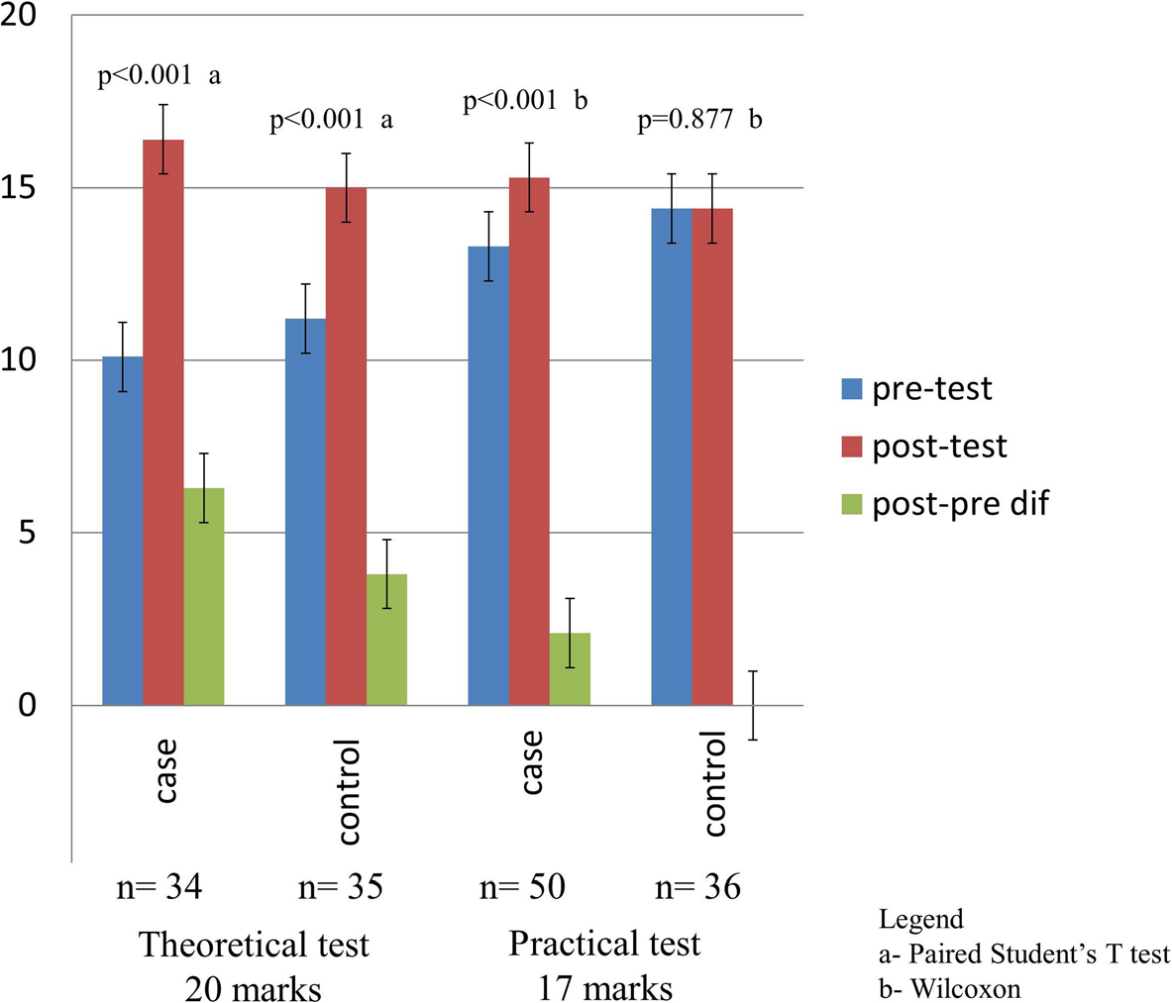
Paired analysis in the theoretical and practical pre- and post-tests of the case and control groups (mean and standard deviation)

The students’ performance (case and control) was also assessed through paired analysis before and after tests (Table 2 and Fig. 2).

**Table 2:** Paired analysis in the theoretical and practical pre- and post-tests between case and control groups

Assessment (maximum)	Group	n	Pre-testx±s	Post-test x ± s	*p* value	Difference between post- and pre-	*p* value of the difference
Theoretical test (20 marks)	Case	*34*	*10,1 ± 3,0*	*16,4 ± 1,7*	*< 0,001* ^*a*^	*6,3 ± 2,8*	*< 0,001* ^*c*^
	Control	*35*	*11,2 ± 2,6*	*15,0 ± 2,3*	*< 0,001* ^*a*^	*3,8 ± 2,5*	
Practical assessment (17 marks)	Case	*50*	*13,3 ± 2,1* ^*e*^	*15,3 ± 1,2* ^*e*^	*< 0,001* ^*b*^	*2,1 ± 2,0* ^*e*^	*< 0,001* ^*d*^
	Control	36	14,4 ± 1,6^e^	14,4 ± 1,4^e^	0,877^b^	0,0 ± 1,6^e^	

x ± s– mean ± standard deviation

^a^Paired Student’s T test

^b^ Wilcoxon signed-rank test

^c^Student’s T test

^d^Mann Whitney

^e^ Nonparametric

The first practical assessment was the practical pre-test and the second one was the practical post-test

The performance of the community in the theoretical pre-test and in the practical assessment was compared with that of all the students (case plus control groups) whereas the theoretical post-test and the variation of the theoretical gain (post-test minus pre-test) were compared separately with the case and control students (Table 3).

**Table 3:** Comparison between the performance of the community and the students

Assessment (maximum)	Students			Community		*p* value
	Group	n	x ± s	n	x ± s	
Theoretical pre-test (20 marks)	All	73	10,6 ± 2,8	228	7,9 ± 3,6^c^	*< 0,001* ^*a*^
Theoretical post-test (20 marks)	Case	34	16,4 ± 1,7	166	13,4 ± 3,2^c^	*< 0,001* ^*a*^
	Control	37	15,0 ± 2,3	166	13,4 ± 3,2^c^	*0,001* ^*a*^
Theoretical variation (post-test – pre-test)	Case	34	6,3 ± 2,8	155	5,4 ± 4,0	0,229^b^
	Control	35	3,8 ± 2,5	155	5,4 ± 4,0	*0,003* ^*b*^
Practical (17 marks)	All	91	13,8 ± 2,0^c,d^	162	11,7 ± 3,2^c^	*< 0,001* ^*a*^

x ± s – mean ± standard deviation

^a^Mann Whitney

^b^Student’s T test

^c^ Nonparametric

^d^1st practical assessment

Of the 240 people in the community, 155 performed both the theoretical pre- and post-tests, with a mean of 7.9 ± 3.6 and 13.3 ± 3.2(maximum = 20), respectively (*p* < 0.001). There was no difference in the theoretical performance of the community that was taught by the case or control group (*p* = 0.113). The same occurred in the practical assessment (*p* = 0.833).

**Fig. 3:**
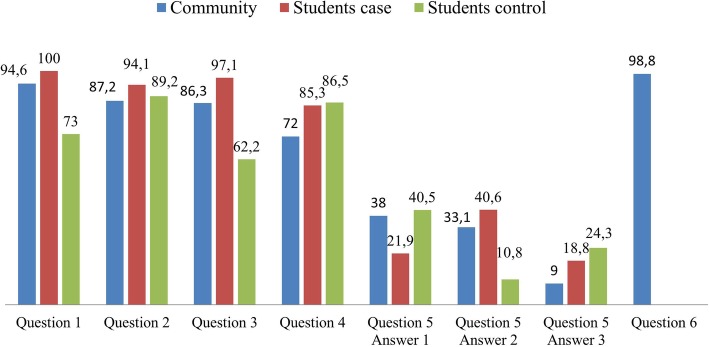
Participants’ positive reactions in relation to activities (%). Question 1: Do you feel able to apply your knowledge about Basic Life Support in an emergency situation? (Y / N). Question 2: Were the lessons enough for your learning? (Y / N). Question 3: After your learning, do you consider yourself capable of teaching others about Basic Life Support? (Y / N). Question 4: What do you think was the most important part of the course? Answer: the practical classes. Question 5: What do you think could be improved in relation to the course?. Answer 1: more practice. Answer 2: increase the workload. Answer 3: more mannequins. Question 6: Would you take other courses delivered by university students? (Y / N)

The perception of the participants, community and students, after the activities, regarding their own use and related experiences, are demonstrated in Fig. 3. The students’ results are divided into the case and control groups. The first responded after the teaching experience and the second before it.

## Discussion

The students in the case group obtained cognitive and skills performance superior to that of the control group, measured by the theoretical and practical pre- and post-tests. This corroborates the studies that also used medical students to teach BLS techniques [23, 24]. However, in general, the studies with medical students acting as BLS trainers do not measure the impact of the didactic activity [21, 22, 35]. In the literature, only one study [23] presented a similar methodology with students teaching children and measuring the gain of knowledge and skills. Nevertheless, in that study, the case group had to teach after attending a BLS course, while the control group neither had to teach nor attend the BLS course. The superior gain in the performance of the case group may have been both because of the course and the teaching experience. This possible bias was avoided in the present study on account of the methodological design.

Here it was possible to measure the gain of knowledge and skills of the learning by teaching and to reach a higher level within the Kirkpatrick scale, going beyond the assessment of the reaction after the activity [36]. A greater retention of knowledge and skills with the teaching practice was demonstrated, resulting in a significant increase in the case group learning, as verified by the difference obtained in the practical assessments.

Besides this, the two groups of students have benefited from the training experiences, behaved similarly in self-assessment, and were equally well assessed by the teacher who did not participate in the process.

During the research, there were reports of community participants who, in a real situation of cardiopulmonary arrest, were able to employ BLS. Although those were occasional reports, it is believed they were a sign that the intervention altered the way they act, which would correspond to Kirkpatrick’s level of action, though this was not the focus of this study.

Being a usual curricular activity for the students, it was possible to maintain a homogeneous group, with the majority of the participants present in the stages and assessments [24]. However, in the community group, although they were volunteers and were released from their usual work to participate, their attendance oscillated at different stages. Three different days of activities greatly contributed to absenteeism. The posters, the course booklet and the certificate were motivational elements used to attract the community, but personal factors related to work and holidays prevented the maintenance of the same number of individuals at all stages. Variations in the attendance of the members are common facts in studies involving human beings [23].

Due to difficulties related to the school calendar, which changed as a result of holidays and strikes, theoretical and practical re-assessments did not occur in an equal period of days among the five participating classes. The three classes in the case group were re-assessed between 60 and 75 days of the first assessment, and the two classes of the control group between 50 and 70 days. However, the authors did not consider that this difference could have affected the groups.

The students’ performance in the second assessment, theoretical or practical, was higher than the first one, except in the practical assessment of the control group. This shows that the theoretical and simulated classroom training were sufficient to improve students’ theoretical performance (cognitive). However, when the act of teaching was associated the case group achieved superior performance. As the assessment instrument was the same in the theoretical pre- and post-test, familiarity with the instrument itself and learning from one’s own mistakes may have been a way of improving performance, regardless of the training received in the classroom or the act of teaching. On the other hand, the same was not observed in the practical assessment. The control group, who had received the preparation to teach while they prepared posters and booklets [10], presented similar performance between the first and second practical assessments, although both used the same simulated station followed by feedback, without improving resuscitation skills [37, 38]. The case group presented a much better performance in the second practical assessment. The best explanation for this difference between the groups is the intervention performed: teaching BLS to others. It is interesting to highlight that the feedback was not shown as an effective tool capable of improving the skill, even when well delivered (based on direct observation, immediately after the assessment, beginning with the learner’s self-assessment, focusing on performance) [31].

At the same time, the study served to disseminate BLS to the members of the community. Other studies have already demonstrated the potential multiplier of teaching BLS to teachers and health professionals [39, 40]. The community, with professionals of different school levels, but predominantly secondary school level, presented a lower performance in the theoretical pre-test, theoretical post-test and practical assessment compared to the medical students, maybe because of their different educational background. However, in the theoretical range variation (post-test minus pre-test), there was no difference between community and case group. This is probably due to the good performance in the post-test of the case and the poor performance in the community pre-test, affecting the range of test variation. It should be noted that students had more classes and training than community members, with access to the simulation laboratory and the monitors’ help during their classroom training, which may have contributed to their performance. Another reason is that students’ scores were part of the curricular unit (summative assessment). A certificate of participation was offered for members of the community, regardless of their performance, which would fatally increase absenteeism in the activities, compromising one of the foundations of the study, which was the promotion and dissemination of resuscitation techniques. The theoretical performance of the community increased after the course, demonstrating that students were efficient in the teaching task. There was no difference between the performances of the professionals who were taught by the case or control groups.

The perceptions related to the activities performed were assessed by a questionnaire applied concomitantly to the theoretical post-test, thus, at different times in the two groups. In the case group, this questionnaire was applied after teaching and in the control group, before. As for the ability to apply knowledge and skills about BLS in a real situation, 94.6% of community participants felt capable after attending the course, as did 100% of students in the case group. In the control group, with only classroom learning and answering the question about 60 days after learning BLS and before teaching, only 73% felt able to perform these techniques. The degree of confidence in the ability to perform BLS is related to the way it is taught [41]. As for the sufficiency of the lessons for learning, more than 87% of the members of the three groups answered it positively. Concerning the perception of the ability to teach others, there was a great difference between case (97.1%) and control (62.2%), since the first one answered after and the second before teaching. Teaching has brought more security and confidence to students. The three groups pointed out the practical classes as the most important for the course, highlighting the simulation with the use of mannequins. The community, being asked if they would attend other courses performed by the medical students, answered with 98.8%. in the affirmative. These data corroborate other studies that have used students in the role of teachers with good acceptance by themselves and by the target audience [28, 35, 42]. It also demonstrates the lack of training courses for local health professionals, as well as the social role that the students can represent, as agents of transformation of the environment where they are involved.

It seems logical to think that the improvement in performance and knowledge found after the teaching of Cardiopulmonary Resuscitation can be extended to a greater use of this teaching methodology in Medical Education, extrapolating its use to a potential improvement in the performance of other medical skills, showing similar results to those presented.

## Conclusions

The BLS teaching activity has proven to be a viable and effective method to increase students’ knowledge and skills, more effectively than lessons associated with feedback simulation. This allowed what was learned in the simulation laboratory by the medical students to be retained in a more efficient way. The feedback after the practical assessment, without the act of teaching, has not been enough to improve BLS skills. Students were able to play a social role by disseminating and replicating BLS knowledge to health professionals and laymen who were able to acquire knowledge and skills through the simulations. The university, going beyond its walls and interacting with the community, plays its part in social accountability.

## Abbreviations

ACLS: Advanced Cardiovascular Life Support
AED: Automatic External Defibrillator
BLS: Basic Life Support

## References

[1] Pereira LL, Santos LMP, Santos W, Oliveira A, Rattner D (2016). Mais Médicos program: provision of medical doctors in rural, remote and socially vulnerable areas of Brazil, 2013-2014. Rural Remote Health.

[2] Santos LMP, Oliveira A, Trindade JS, Barreto IC, Palmeira PA, Comes Y (2017). Implementation research: towards universal health coverage with more doctors in Brazil. Bull World Health Organ [Internet].

[3] Bestetti RB, Couto LB, Romão GS, Araújo GT, Restini CBA (2014). Contextual considerations in implementing problem-based learning approaches in a Brazilian medical curriculum: the UNAERP experience. Med Educ Online.

[4] Duffy TP (2011). The Flexner report - 100 years later. Yale J Biol Med.

[5] Michael J (2006). Where’s the evidence that active learning works?. Adv Physiol Educ.

[6] Mennin S, Petroni-Mennin R (2006). Community-based medical education. Clin Teach.

[7] Hays R (2007). Community-oriented medical education. Teach Teach Educ.

[8] Taylor DCMM, Hamdy H (2013). Adult learning theories: implications for learning and teaching in medical education: AMEE guide no. 83. Med Teach.

[9] Palis A, Quiros P (2014). Adult learning principles and presentation pearls. Middle East Afr J Ophthalmol [Internet].

[10] Fiorella L, Mayer RE (2013). The relative benefits of learning by teaching and teaching expectancy. Contemp Educ Psychol [Internet]. Elsevier Inc.

[11] Grzega J, Schöner M (2008). The didactic model LdL (Lernen durch Lehren) as a way of preparing students for communication in a knowledge society. J Educ Teach.

[12] Peets AD, Coderre S, Wright B, Jenkins D, Burak K, Leskosky S (2009). Involvement in teaching improves learning in medical students: a randomized cross-over study. BMC Med Educ.

[13] Miller GE (1990). The assessment of clinical skills/competence/performance. Acad Med [Internet].

[14] Kleinman ME, Brennan EE, Goldberger ZD, Swor RA, Terry M, Bobrow BJ (2015). Part 5: adult basic life support and cardiopulmonary resuscitation quality: 2015 American Heart Association guidelines update for cardiopulmonary resuscitation and emergency cardiovascular care. Circulation..

[15] Friesen J, Patterson D, Munjal K (2015). Cardiopulmonary Resuscitation in Resource-limited Health Systems-Considerations for Training and Delivery. Prehospital Disaster Med [Internet].

[16] Bewley WL, O’Neil HF (2013). Evaluation of medical simulations. Mil Med [Internet].

[17] Santos SV, Margarido MRRA, Caires IS, Santos RAN, Souza SG, Souza JMA (2015). Basic life support knowledge of first-year university students from Brazil. Brazilian J Med Biol Res [Internet].

[18] Hasselqvist-Ax I, Riva G, Herlitz J, Rosenqvist M, Hollenberg J, Nordberg P (2015). Early cardiopulmonary resuscitation in out-of-hospital cardiac arrest. N Engl J Med [Internet].

[19] Madl C, Holzer M (2004). Brain function after resuscitation from cardiac arrest. Curr Opin Crit Care.

[20] Bakke HK, Steinvik T, Angell J, Wisborg T. A nationwide survey of first aid training and encounters in Norway. BMC Emerg Med [Internet]. BMC Emergency Medicine; 2016;17:6. 10.1186/s12873-017-0116-7 PMC5322636 28228110

[21] Fraga GP, Carvalho RB, Hirano ES, Bollela VR. Basic life support: medical students learning by teaching. Med Educ [Internet]. 2012;46:1105–1105. 10.1111/medu.12026 23078692

[22] Ribeiro LG, Germano R, Menezes PL, Schmidt A, Pazin-Filho A (2013). Medical students teaching cardiopulmonary resuscitation to middle school Brazilian students. Arq Bras Cardiol [Internet].

[23] Beck S, Meier-Klages V, Michaelis M, Sehner S, Harendza S, Zöllner C (2016). Teaching school children basic life support improves teaching and basic life support skills of medical students: A randomised, controlled trial. Resuscitation [Internet].

[24] Breckwoldt J, Beetz D, Schnitzer L, Waskow C, Arntz HR, Weimann J (2007). Medical students teaching basic life support to school children as a required element of medical education: a randomised controlled study comparing three different approaches to fifth year medical training in emergency medicine. Resuscitation..

[25] Wen LS, Greysen SR, Keszthelyi D, Bracero J, De Roos P (2011). Social accountability in health professionals’ training. Lancet..

[26] Woollard RF, Fcfp C. Social accountability and accreditation in the future of medical education for the 21st century. Heal San Fr. 2010:1–25.

[27] Lee JH, Cho Y, Kang KH, Cho GC, Song KJ, Lee CH. The effect of the duration of basic life support training on the learners ’ cardiopulmonary and automated external defibrillator skills. Biomed Res Int. 2016:1–7. 10.1155/2016/2420568 PMC4978818 27529066

[28] Perkins GD, Hulme J, Shore HR, Bion JF (1999). Basic life support training for health care students. Resuscitation..

[29] Veloso SG, Pereira GS, de Faria RMD, Senger MH (2016). Basic life support: students teaching community health workers. Med Educ.

[30] Bjørnshave K, Krogh LQ, Hansen SB, Nebsbjerg MA, Thim T, Løfgren B. Teaching basic life support with an automated external defibrillator using the two-stage or the four-stage teaching technique. Eur J Emerg Med [Internet]. 2016:1–7. 10.1097/MEJ.0000000000000410 27203452

[31] Ramani S, Krackov SK (2012). Twelve tips for giving feedback effectively in the clinical environment. Med Teach..

[32] Mahling M, Münch A, Schenk S, Volkert S, Rein A, Teichner U (2014). Basic life support is effectively taught in groups of three, five and eight medical students: a prospective, randomized study. BMC Med Educ [Internet].

[33] Walzak A, Bacchus M, Schaefer JP, Zarnke K, Glow J, Brass C (2015). Diagnosing technical competence in six bedside procedures: comparing checklists and a global rating scale in the assessment of resident performance. Acad Med.

[34] Evans AW, McKenna C, Oliver M (2002). Self-assessment in medical practice. J R Soc Med.

[35] Robak O, Kulnig J, Sterz F, Uray T, Haugk M, Kliegel A (2006). CPR in medical schools: learning by teaching BLS to sudden cardiac death survivors--a promising strategy for medical students?. BMC Med Educ [Internet].

[36] Frye AWNNW, Hemmer PA (2012). Program evaluation models and related theories: AMEE guide no. 67. Med Teach [Internet].

[37] Nolan JP (2014). High-quality cardiopulmonary resuscitation. Curr Opin Crit Care.

[38] Li Q, Hua ZR, Liu J, Lin J, Ma EL, Liang P (2013). Pre-training evaluation and feedback improved skills retention of basic life support in medical students. Resuscitation.

[39] Toner P, Connolly M, Laverty L, McGrath P, Connolly D, McCluskey DR (2007). Teaching basic life support to school children using medical students and teachers in a “peer-training” model-results of the “ABC for life” programme. Resuscitation..

[40] Levett-Jones T, Lapkin S (2014). A systematic review of the effectiveness of simulation debriefing in health professional education. Nurse Educ today [internet]. Elsevier Ltd.

[41] Lami M, Nair P, Gadhvi K (2016). Improving basic life support training for medical students. Adv Med Educ Pract [Internet].

[42] Harvey PR, Higenbottam CV, Owen A, Hulme J, Bion JF (2012). Peer-led training and assessment in basic life support for healthcare students: Synthesis of literature review and fifteen years practical experience. Resuscitation [Internet].

